# ‘Don’t lose it on the bus!’: Casting the normative biosexual citizen in early Scottish pre-exposure prophylaxis provision

**DOI:** 10.1111/1467-9566.13632

**Published:** 2023-03-15

**Authors:** Ingrid Young, Nicola Boydell

**Affiliations:** Centre for Biomedicine, Self and Society, Usher Institute, University of Edinburgh, Edinburgh, UK

**Keywords:** HIV/AIDS, inequalities, pre-exposure prophylaxis (PrEP), sexual health

## Abstract

The introduction of HIV pre-exposure prophylaxis (PrEP) raises important questions around how new biotechnologies are negotiated within contemporary settings and how they can shape the moral governance of biocitizens, or as we explore, biosexual citizens. This article draws on qualitative interviews and focus groups to consider how the normative biosexual citizen was cast at the start of provision in Scotland by clinical and community practitioners. Our findings show how practitioners navigated ideas around who was deserving of support and access to PrEP in the context of limited resources, interpreted what legitimate risk narratives might look like for different groups and translated particular gendered, sexualised and racialised risk profiles in the context of PrEP provision. This draws attention to how normative biosexual citizenship was not determined through meeting a set of clinical criteria and adhering to a prophylaxis regime but cast through ongoing negotiations with clinical and community practitioners in relation to normative ideas of essential care, constrained resources, risk narratives and gendered and racialised bodies. Our research indicates how access to PrEP will continue to demand particular enactments of normative biosexual citizenship that may well be at odds with the experiences and needs of communities affected by HIV.

## Introduction

Pre-exposure prophylaxis (PrEP)—where HIV-negative individuals take existing HIV treatment to prevent HIV acquisition—has taken the HIV prevention world by storm. Celebrated as a key tool to bring an ‘end to AIDS’ (Bernays et al., 2021), the use of HIV treatment as a prophylaxis has dramatically changed HIV prevention globally. The introduction of PrEP also reveals the complexities in the implementation of novel biotechnologies throughout health systems, especially the use of pharmaceuticals for public health (Brandt, 1987; Greene et al., 2016). Although the turn to biotechnologies as tools for health is not new, it is important to consider how new biotechnologies are negotiated within contemporary settings, paying particular attention to if and how they can disrupt and/or be integrated into existing systems. Moreover, we need to be attentive to how new biotechnologies can shape the moral governance of biocitizens (Johnson et al., 2018), or, as we will go onto explore, biosexual citizens (Epstein, 2018). It is important, therefore, to consider how PrEP is imagined *by* health practitioners and *for* patients and how this shapes its availability and access across clinical and community settings. Thus, our inquiry explores how PrEP has been imagined and translated into and across diverse communities in Scotland.

Scotland was one of the first countries in the UK and internationally to offer PrEP through a publicly funded health service; PrEP has been available through Scottish National Health Service (NHS) sexual health services since July 2017 (Young, 2021). Existing policy frameworks in Scotland enabled policymakers and practitioners to circumvent some of the obstacles to PrEP provision in other nations of the UK, most notably England (Dodds, 2021; Paparini, 2021). Scotland was able to capitalise on PrEP momentum as a result of clinical trial evidence and relevant governance mechanisms, resulting in favourable policy decisions and, ultimately, provision (Young, 2021). However, initial provision of PrEP came at a high cost for the Scottish NHS; for the first 5 months of provision, PrEP was at its highest (branded) price, and no additional financial support was made available from the health budget. Resources were re-allocated from within existing health board budgets to pay for both the drugs and for supplementary clinics within already overstretched services. As a relatively early provider of PrEP globally through state-funded health systems, we are particularly interested in how not only the *provision* of PrEP manifests in a publicly funded health system but how PrEP—and PrEP users—are made, in part, by the very system that provides it. What expectations might this create, and who and what might this exclude?

There has been important work showing how the parameters of PrEP use—and specific constructions of the imagined PrEP user—have been narrowly conceived. This work has shown how PrEP trials, demonstration studies and even strategic rollout of PrEP in the UK (Dodds, 2021), Europe (Demart & Gerard, 2022) and Australia (Smith et al., 2022a) contributed to specific imagined PrEP users. This framing, largely supported by existing structures of sexual health services through which PrEP was delivered, has limited possibilities of who else might benefit from PrEP by reinforcing existing inequalities in access (Dodds, 2021). Here, we consider how clinical health providers and community health support workers anticipated and responded to existing and potential PrEP users at the point of PrEP implementation in Scotland. As we explore, the casting of PrEP users as normative biosexual citizens is more than simply assessing eligibility against clinical guidelines or informing communities about the availability of PrEP; health practitioners who interpret criteria and prescribe PrEP play a key role in enabling access to and shaping what PrEP is, playing their part in constituting PrEP users and casting normative PrEP biosexual citizens in the process. In this article, we explore PrEP as a citizenship project and consider how the normative biosexual citizen was cast at the start of provision in Scotland by clinical and community health practitioners. This exploration allows us to understand the longer-term implications of how PrEP is positioned within the health system, for whom it is accessible and how the orientation of PrEP for imagined biosexual citizens is constituted through its implementation.

## Casting Normative Biosexual Citizenship

The introduction and use of novel technologies in health are well-studied across medical sociology and feminist science and technology studies (STS) (Johnson, 2017; Nelson, 2017; Peterson et al., 2017), unpacking the ways in which they can disrupt and shape our very ideas of health and influence our health practices. Medical sociologists have sought to understand how professionals and patients interact through and are shaped by medical technologies, how these interactions shape new meanings of health and how categories of patienthood, humanity, disease, risk and health are forged (Caspar & Morrison, 2010). Much of this work has sought to understand not only how technologies have become tools of medicalisation but how the increasing centrality of technologies in health are part of a shift to biomedicalisation (Bell & Figert, 2015; Clarke et al., 2010). Clarke et al. (2010) document how this reflects a shift in medicine from exerting control over bodies and particular conditions to how biomedicine has become increasingly ‘techno-scientifically constituted’, now capable of transforming bodies, creating new possibilities. Thus, consideration of how novel technologies have the potential to shape and change ideas of health, patienthood and self-governance within and across health systems—and how local dynamics of health inequities might shape this—is of critical importance (Bell & Figert, 2015).

Biocitizenship—a key component of biomedicalisation (Clarke et al., 2010)—describes how self-governance shapes patienthood, health practices and negotiations with state institutions. It has been widely explored across sociological and related literature (Johnson et al., 2018; Nguyen, 2010; Paparini & Rhodes, 2016; Petryna, 2002; Rose, 2007; Young et al., 2019). Applied across a range of health conditions, communities and technoscientific systems, biocitizenship as a theoretical concept establishes how rights *and* responsibilities govern the health practices of communities; there is an expectation of both a right *to* healthcare, as well a responsibility to care *for* oneself and others (Nguyen, 2010; Petryna, 2002; Young et al., 2019). The concept has been widely used to explore community experiences of navigating a complex health landscape to care for themselves. Research has highlighted the work done by communities to be ‘good’ biocitizens, as well as the stratification of biocitizenship and its broader implications in the possibilities of negotiating structural and intersectional inequalities, such as gender and sexual identities, race and location (Kolopenuk, 2020; Paparini & Rhodes, 2016; Rinaldi et al., 2017; Russell et al., 2016; Squire, 2010; Teixeira & Christina Dias, 2015; Young et al., 2019).

In this article, we draw on the concept of *biosexual* citizenship, by which we mean the entanglement of specific sexual identities, communities and practices with biocitizenship and how this shapes the intersection between rights and responsibilities for both individuals and communities (Epstein, 2018; Jones et al., 2020). Biosexual citizenship is a useful concept to consider not only how self-governance shapes health practice in relation to biomedicine and pharmaceutical prevention but also how it explicitly draws in and negotiates with gendered and sexual identities, practices and rights. Epstein’s introduction of the concept examines how pleasures and risks associated with sexuality figure in biomedicine and public health and the way this shapes and makes possible sexual rights and responsibilities (Epstein, 2018). Elsewhere, we have explored how particular enactments of biosexual citizenship have been constitutive of PrEP activism in the UK; PrEP activist community demands for resource distribution from the state are accompanied by enactments of biosexual citizenship grounded in responsibilities to the health and rights of queer communities and the possibilities of ‘self-care’ (Jones et al., 2020). In this way, biosexual citizenship enables us to consider not only how PrEP-related health practices and health care are grounded in notions of ‘responsible’ conduct and expectations from state institutions but how they are embedded within a gendered and sexualised landscape, invoking particular expectations around sexual health, rights and practices.

We suggest, along with others, that it is important to consider the health and wider social infrastructure in which communities (attempt to) gain access to PrEP provision. Where biosexual citizenship allows us to explore the intersections of gender/sexuality, PrEP access and health systems requirements, we also must be attentive to how expectations of citizenship manifest. Johnson et al. (2018) in their consideration of biocitizenship warn of the potential risks associated with the discourse of good or model ‘biocitizens’:

Although Biocitizenship has proved to be a powerful descriptive term, when it takes on a normative cast—that is, when the biocitizen becomes something we ought to be—things become more complicated. Underlying this affirmative discourse is an image of the model biocitizen, who is assumed to be a rational, autonomous actor, health and able-bodied (or, importantly, wants to be) and has some measure of class privilege. When yoked to biomedicine, and when biomedicine is tied to state and corporate interests, the biological citizen thus becomes a much more troubling figure.

(Johnson et al., 2018)

Here Johnson et al. draw our attention to how the citizenship discourse of self-governance in relation to rights and responsibilities can (easily) become a way to shape access to and expectations of appropriate health practices, implicated in state and corporate interests and tied to normative social structures. We suggest it is important to consider if, how and where this casting—of *biosexual* citizens—occurs. In the remainder of this article, we ask how the normative casting of biosexual citizenship manifests in and by those who are providing access to PrEP. Moreover, we explore how this entanglement of expectation shapes access. In other words, we will seek to explore how biosexual citizenship plays a role in clinical and community interactions and what the implications are for communities in Scotland.

## Methods

The analysis presented in this article was developed as part of a research study *Developing HIV Literacy*. The aim of the study was threefold; to understand the experiences of and challenges in providing PrEP in Scottish NHS sexual health services and relatedly within community support settings; to identify and explore the HIV literacy challenges of PrEP implementation, including the translation of existing clinical PrEP information within and across diverse communities who may benefit from PrEP; and to understand how PrEP was imagined as a tool for HIV prevention by health practitioners and the implications for provision (Young & Valiotis, 2020).

Drawing on well-established qualitative methods (Mason, 2017), IY undertook in-depth interviews and focus groups (Barbour, 2018) with clinical and community health practitioners who were involved in supporting the dissemination of PrEP information and support of PrEP use. Clinical participants were recruited through existing health promotion networks in NHS boards across urban and rural Scotland. Community participants were recruited from third sector organisations who had been involved in prevention and/or care for communities affected by HIV and LGBTQ wellbeing and who were directly involved—in some way—in PrEP awareness and support work.

Phase 1 (May 2017) data collection was undertaken with community practitioners in anticipation of PrEP provision and discussions centred around the key concerns in communicating PrEP; identifying key communities; and barriers to PrEP delivery and support. Phase 2 data collection (August–October 2017) took place following NHS PrEP provision with community and clinical practitioners. These interviews explored experiences of providing community and/or clinical support for PrEP now available through NHS sexual health services. The study recruited participants via email through existing formal and informal health promotion networks across Scotland and through known third sector organisations working in HIV prevention. All study participants were provided with information about the research and gave consent for participation. Interviews and focus groups ranged from 60 to 90 min, were undertaken in person (with one telephone interview), were digitally recorded, transcribed and anonymised. This study received Research Ethics approval from the Usher Research Ethics Group, University of Edinburgh.

In total, 32 participants took part in the study: 19 community practitioners and 13 clinical practitioners. Participants included practitioners who worked with those affected by HIV, namely gay and bisexual men, Black African communities and other racialised communities, primarily but not exclusively located in Scotland. Over half of all participants identified as being from the communities with whom they worked. Some participants were involved in advocating for PrEP within Scotland, including a very small number who contributed to policy development and in some cases strategic decisions. However, most were not involved in policy decisions, assessment of cost-effectiveness or evaluation of PrEP at the time the data collection took place.

Analysis was an iterative process: IY undertook a preliminary analysis to identify key issues and map out themes; these themes were then explored with the wider Developing HIV Literacy project academic, clinical and community partners through two half-day workshops. These workshops consolidated deductive themes driven by the aims of the wider Developing HIV Literacy project but also identified further issues around the blurring of boundaries between ‘literacy’ and service provision. Identification and refinement of themes for this analysis drew on these issues, and analysis was supported by extensive discussions between authors. Both authors then undertook a systematic coding and analysis of the data, initially individually and then together, to identify and expand on the key themes presented in this article. Initial coding was done using NVivo 10 qualitative data software by NB and was further supplemented and refined by ongoing discussions and reflections between the two authors. See (Young & Valiotis, 2020) for further details on methodologies, analysis and study sample.

### Findings

We present findings in three broad themes. *Costs, burdens and responsible use of resources* consider how practitioners positioned the cost of PrEP and availability of resources against the needs of ‘other’ patients and services. We then explore how clinical and community practitioners worked to understand and *shape PrEP narratives* and where and how these were in tension. Finally, we consider intersectional inequalities in *who doesn*’*t fit* and the race and gendered implications for providers and for those who might benefit from PrEP.

### Costs, burdens and responsible use of resources

As outlined above, the introduction of PrEP came with significant costs. Although there was a move away from branded Truvada to a generic—and therefore much cheaper—version of PrEP by the end of 2017, the initial high costs and increased demands on existing services was a critical issue for health services and for our participants. Perhaps unsurprisingly, anxieties about the responsible use of NHS funds and ‘non-essential’ provision of PrEP proliferated discussions in our interviews with clinical practitioners. Many clinical practitioners spoke extensively about the costs of PrEP and their anxieties about the increased burden PrEP provision and services were having on an already stretched health service. One participant described how costs were foremost in their mind during PrEP consultations, especially where people presenting - predominantly cis-gay men^1^ perceived to be relatively affluent—reported previously buying it online themselves.

***Respondent 3*:** What we’re being inundated with at the moment is the well-educated, well-to-do gay men who could afford to pay £40 a month, and I feel slightly peculiar handing out three months packs, and I say to them “this is £1200 worth of medication, don’t lose it on the bus!”

***Respondent 1*:** I’ve got to author[ize] it, I see the bill coming in and I’m just horrified, the first one that came in was £26,000 and that’s every week.

***Respondent 2*:** It’s going to be expensive isn’t it?

***Interviewer*:** So would you prefer that they continued to source it online?

***Respondent 3*:** If they’re, you know, the ones who come in suits who’ve been buying it online and who just kinda say “now I can get it free”, I say “well it’s not free, this is costing the country £1200, this is £1200 worth, you’ve got to think about it in that kinda way”. I mean, as somebody working in, you know, I question the government decision to not wait until it was all available generically, it just seems a highly illogical kind of thing, very costly thing in a country that’s going down the gurgle hole with no money.

(Clinical Practitioners Focus Group)

In emphasising the cost of PrEP to the NHS and reiterating to patients that they shouldn’t ‘*lose* [their PrEP] *on the bus*’, this extract highlights the ways in which expectations of patient responsibility for management of—or even demand for—drugs is entwined with wider ideas around who is deserving of NHS provision and what is deemed essential health care. We see here a concern not only about the costs to the NHS for the medication but also a judgement that people who appear to be well-resourced—signalled by wearing ‘*suits*’—and/or who have already secured access privately should not be making claims on an already stretched NHS. This, participants insisted, was not about individuals or groups but was a question of equity within a wider, underfunded health system and at a time when the cost of PrEP to health services was high.

While not all clinical practitioners expressed concerns about costs in this way, some relayed discussions they had with patients about the responsible use of NHS resources, demonstrating how perceived PrEP costs were a concern shared by both clinical practitioners and (some) PrEP users. One participant described a conversation with a gay man who had been self-sourcing PrEP and who was reluctant to switch to NHS provision:

Well, some are eligible to switch [from self-sourcing] and some just go straight away, and some are in a real dilemma over it. I had a conversation just last Wednesday night with a guy who said “I can afford it, I really don’t think I should have it but I don’t have any children and I pay loads of taxes” [Laughter] “and then I think I should just be able to have something!” [Laughter]…I said “I’m just going to leave it with you, what I suggest is you stay self-sourcing till next time, I’ve documented in your notes, if you decide to switch to NHS that’s fine but I’m not taking you through that one, that’s just for you to work out!” but yeah, you do get people say “I want it but I acknowledge it’s a lifestyle drug and I don’t think the NHS should be paying for it cause I can afford it.”

(Clinical Practitioners Focus Group)

This exchange illustrates how assessing PrEP as a ‘*lifestyle drug*’ plays an important role in shaping understandings of the responsible use of NHS resources. Weighing up contributions *to* a health system (paying taxes and having no children) and demands *from* this system (NHS provision of an expensive drug) here is further complicated by framing PrEP as a non-essential medication, or at least one that can be sourced elsewhere. We see here tensions clinical practitioners faced not only with assessing clinical eligibility but also being drawn into negotiations of what socially legitimate biosexual citizenship might look like in the context of resource scarcity (See Keogh 2017; Young et al., 2019).

Drug costs were only one part of navigating what PrEP meant for equitable resource allocation for participants. A vocal minority of clinical practitioners expressed concerns about the detrimental impact of the provision, describing what they deemed to be the unfair prioritisation of PrEP over other services. Making space to provide PrEP services within already oversubscribed sexual health clinics, especially those offering integrated sexual and reproductive health services, sometimes resulted in the need to triage or prioritise certain patients. One participant explained what they saw as the discrepancy between how this translated into the experiences of PrEP patients—primarily viewed as cis-gay men—and other patients—implicitly understood as cis-women—who sought other services:

Because you know, if you’re wanting PrEP you get seen straight away, but if you want a coil fitted then it’s about a couple of months….and you’re kinda like well that person’s got to use condoms till they come in for their coil and then if you need counselling for sexual abuse it’s a year, so you’re kind of like is the priority, is that really right? Should it be not maybe wait slightly longer for PrEP and less for counselling or something? You know, so it just seems very, you know…I suppose there’s only a limited amount of appointments.

(Clinical Practitioner Interview)

Here, PrEP services are positioned as exacerbating problems with already stretched services. While this is constructed as a health service issue—‘*only limited amount of appointments*’ —it also suggests that *some* clinical practitioner expectations of those using services are that they ought to be deserving and responsible biosexual citizens, only seeking out *essential* services and medication so as not to take away from other ‘deserving’ patients. This reflects how PrEP services—and in particular PrEP patients—were cast by *some* clinical practitioners as scapegoats for accessing non-essential resources in overstretched services. In keeping with the idea that PrEP is a ‘lifestyle’ drug, and therefore non-essential, and in contrast to the essential need for contraception (coil) or counselling for sexual abuse, these sentiments echo opposition to making PrEP available in England, where the Department of Health pitted gay men’s demands for PrEP *against* cancer and paediatric care (Henderson, 2018). This stance has been read by some as homophobic (Jones et al., 2020; Mowlabocous, 2019) in that it positions PrEP recipients—presumed gay men—as less deserving of access to expensive health technologies than other, understood to be more deserving patients.

While community practitioners had less to say about costs to the NHS, the issue was raised in discussions they had with clinical partners. One participant explained how they were told by an HIV practitioner not only how much PrEP had cost the health service but also that this was framed this in relation to staffing of HIV clinics and the costs of services for people living with HIV:

***Respondent*:** … that was up to two weeks ago, the amount of money that PrEP has cost the NHS in [name of city] is three full time members of staff and a clinic for a year. That’s how much she says that they would’ve saved or they could’ve used elsewhere.

***Interviewer*:** So that’s what the prescriptions have cost them.

***Respondent*:** Mm hmm, three full time members of staff and a clinic for a year.

(Community Practitioner Interview)

Building on the idea that PrEP services take away from other, equally important health provision, the suggestion that these funds ‘*could*’*ve [been] used elsewhere*’ effectively pits PrEP users *against* people living with HIV. Although budgetary decisions about HIV prevention and care may not be so cut and dried, the framing of scarce resources with a community practitioner who described themselves as advocating for PrEP is notable. Debates around the high cost of PrEP and strain on existing services expressed by participants raise significant demands on those seeking PrEP. Despite Scottish provision of PrEP being celebrated, the rollout of this new pharmaceutical prevention intervention raised real debates around what essential health care was, and who ought to be accessing it.

### Shaping PrEP narratives

Eligibility for PrEP in Scotland in 2017 was based on meeting at least one of four criteria (HPS & ISD, 2019), intended to identify those at highest risk of HIV. One of the main criterion was having had condomless anal sex with two or more partners in the previous 12 months.^2^ Epidemiologically, this was understood as a *behavioural* marker for heightened HIV risk, and was predominantly applied to cis-gay and bisexual men seeking PrEP. This effectively required the disclosure of multiple sexual partners to secure PrEP via the NHS. Clinicians described how this ‘disclosure’ of risk changed in their clinical conversations with gay and bisexual patients:

It’s definitely improved my discussions about risk and exposure because some guys, I can think of a few who’ve always… obviously there’s two possibilities, one is they want PrEP so they’re telling me about more risk in order to get PrEP. I don’t believe that’s the case in many cases. What seems to be more common is it’s guys who’s coming in “I want to check cause I had an episode of a burst condom” and I’ve had some really nice, really funny ones, there’s a few young guys, particularly one…it was just hilarious, he was just laughing his head off saying “yeah I know I’ve been telling you that for ages and it’s all such crap” [Laughter] and so we had a conversation about the truth which was just amazing, you know, he’d had six or eight unprotected sex partners since the Friday and it was Wednesday, you know, and usually he came and said “oh I had a burst condom three weeks ago, can I have a check up”! [Laughter]… and so then that leads to discussion about where and about sauna and about self-esteem and, yeah, the disclosures have never been a bad thing. So it’s always been good and all that improves your skills in terms of just being able to ask about it.

(Clinical Practitioners Focus Group)

Here we see how the shift in clinical conversations appears to reflect a gauging of what is an acceptable or even necessary risk narrative—or clinical ‘script’—to access services or support. The ‘*burst condom*’ narrative, previously seen as sufficient to access testing and support, is expanded to an ‘open’ discussion of multiple sexual partners. That clinical practitioners reflected on the possibilities that PrEP opened up for gay and bisexual men to introduce and/or expand on their ‘actual’ risk practices and their own skills in discussing risk practices with patients highlights the potential impact of PrEP on clinical encounters and expectations of potential PrEP users. Where previously discussions of risk were described as curtailed, or edited to provide just enough to secure support, the introduction of a pharmaceutical technology that demands a narrative of increased risk practice points to the early shaping of what might become normative PrEP clinical narratives. These findings echo experiences of practitioners reported elsewhere (Nichols & Rosengarten, 2020; Smith et al., 2022b).

A minority of clinical practitioners raised concerns about how PrEP might facilitate problematic—or potentially untrue—risk narratives to what they perceived as a well-established system:

***Respondent 2*:** That’s a double-edged sword I think, the eligibility criteria, that’s a double-edged sword…we have all these rehearsed men [saying] “yes I’ve had anal sex with three different people this year and therefore I’m eligible” and so they’re just kinda rehearsed. Why do we have eligibility criteria? It’s a total waste of time because they already know what the criteria are.

[…]

***Respondent 3*:** I’ve been saying for the last week or two, actually, I think there’s a lot of similarities between PrEP for men and the way the combined pill was for women in the sixties. It was very much regulated you had to be married or about to get married and you had to go to a special clinic to get it and you got it in really small amounts and things, my hope is that eventually we’ll just be giving it out, you know, and they’ll get a year’s supply and they’ll come and get a yearly check and that’s it, you know, but at the moment the way it is I think the potential for men to have rehearsed their eligibility criteria for it is definitely there and who are we to deny them it frankly, you know.

(Clinical Practitioners Focus Group)

The problem as presented—that patients who are informed of what they need to say or do to access PrEP—according to the first participant in this extract suggests that ‘real risk’ should be assessed by a clinician and not rehearsed by patients themselves. PrEP is seen as a clinical intervention that is guided by clinical expertise, rather than knowledge of eligibility criteria: it ought to be based on a *clinical* assessment and not risk-assessment by potential PrEP users themselves. However, this position was challenged by another participant. Rather than restricting access to PrEP, the second speaker suggests that knowledge of clinically acceptable risk narratives for PrEP are not a problem and should instead be seen as a stepping stone to less prescriptive provision of PrEP in the future. This exchange reflects tensions identified across the study in how to effectively identify people who would benefit from PrEP based on their risk narratives and/or compliance with eligibility criteria in the context of a resource-limited setting. We see here how PrEP candidacy is understood by clinical practitioners through narratives of risk, but the validation or recognition of these narratives are importantly shaped by ideas around who is deemed to be clinically deserving.

In the context of perceived clinical ‘policing’ of PrEP, community practitioners in this study described their role in helping shape PrEP narratives to successfully access PrEP in clinical settings. One community practitioner who worked with gay and bisexual men recounted how he advised community members on how to more effectively access PrEP:

***Respondent*:** It sounds bad but I do tell them, you know, you’ve got to really beef it up, you really do […] I would say to them, you know, you’ve got to say things along the lines of, you know, like “When I go out on a Saturday night and I get drunk I don’t really think that well and …I wake up and it’s Sunday morning and I’m with a random stranger and I’ve had unprotected sex again!” You know, rather than them saying that they’ve had protected sex if they’re really wanting to get the PrEP. So it just makes me sort of, like, plant the seed of you’ve got to be that wee bit more… sort of like telling a wee tale around the story to make it more worthwhile for them.

***Interviewer*:** Yeah. And why do you do that, why d’you think that?

***Respondent*:** Because they wouldn’t be asking about PrEP in the first place if they didn’t feel they needed it and I feel that if they’re not going to promote themselves enough, push themselves enough then they’re not going to get it, especially when PrEP started being given out in the clinics, it seemed to be less and less people who were being offered it and more disappointment.

(Community Practitioner Interview)

In contrast to the problematisation of ‘rehearsed narratives’ by the clinical practitioner, this community practitioner suggested that people asking for PrEP was reason enough to support them in accessing it. Citing the early experiences of gay and bisexual men seeking, and being refused, access to PrEP, this participant described how PrEP narratives needed to be ‘*beefed up*’ to be convincing to clinicians and demonstrate how PrEP would be ‘*more worthwhile*’. Moreover, the community practitioner’s advice to potential PrEP users about risk narratives supported them in navigating clinician’s behavioural expectations of people at high risk of HIV at a time when PrEP provision was (perceived as) limited.

### Who doesn’t fit?

Up until this point, we have explored how clinical and community practitioners imagined and responded to potential PrEP users in the context of limited resources and determining the ‘right’ PrEP risk narrative. Explorations of these issues by study participants, however, were almost entirely focussed on cis-gay and bisexual men, with no mention of trans or non-binary people. While this continues to reflect (e.g. HPS, 2019) the primary group accessing PrEP in Scotland at the time of this research, it also serves to emphasise those communities—and in particular, those bodies—that were absent from discussions and clinical settings. Available data on NHS provision highlights the stark disparities in access to PrEP by groups other than cis-gay and bisexual men. In the first 2 years of provision, people other than cis-gay and bisexual men made up less than 2% of PrEP users (HPS, 2019). These numbers reflect, in part, the gendered problems with how the Scottish National Sexual Health System (NaSH), a clinical electronic records system, captures information; at the point of provision in July 2017, NaSH did not record trans identity. The introduction of PrEP prompted a change to the system post-2017 (Young, 2021). The lack of an appropriate category within a clinical electronic records system does not explain the very low numbers of trans PrEP users, however, it does demonstrate that Scottish sexual health services were not set up for trans communities. This further emphasises institutional barriers to a gender-inclusive environment and support for trans communities in Scotland identified elsewhere (Maund et al., 2020). Furthermore, there was no discussion by *most* clinical and community participants about the race or ethnicity of PrEP users. The implicit whiteness of imagined PrEP users is also reflected in the Scottish PrEP data, which reports the majority of PrEP users as white, with only 5.7% of users identifying as a non-white identity where race and/or ethnicity was noted (HPS, 2019). Racial disparities in access to and experiences of health services is a well-documented issue across health systems (Bailey et al., 2021; Russell, 2021). However, much like the absence of trans people from discussions and attendance, the silence in relation to race and ethnicity by participants—with some notable exceptions—shapes the anticipation of the normative PrEP biosexual citizen by health systems.

A small minority of participants did raise issues of gender and race in access to PrEP, primar-ily those who worked in community organisations that supported women and people of colour, principally Black African communities living in Scotland. These participants raised concerns that the introduction of PrEP, like many other HIV-prevention interventions, had not considered PrEP users apart from gay and bisexual men. One participant described how they asked staff at a local sexual health clinic about their plans for the introduction of PrEP and brought up the issue of providing PrEP for women:

I’ve been asking this even before it was incepted, I was in my clinic and I was asking the nurse there, he’s a male, and I said “you say you’re very busy, you’re going to be rolling out in July” - I went in June - “you’re going to be rolling out PrEP, have you considered women?” and he looked at me and says “…actually we have not actually thought about it” and he was being honest to me and I thought to myself “what do you mean you have not thought about it?”

(Community Practitioners Focus Group)

What is striking in this extract is the participant’s surprise at the admission to not thinking about women as potential PrEP users. The apparent lack of consideration to the needs of women in this clinical context reflects what many participants conveyed and what was borne out in data on the uptake: that women were not considered candidates for PrEP. In essence, participants who worked with Black African women recounted how there appeared to be no explicit consideration about the provision for or needs of women and ‘other’ communities, casting clear clinical expectations of who would and would not be supported in the PrEP uptake. This absence of women—and, indeed, other gender diverse people—as PrEP users from a clinical context was also notably borne out in much publicity surrounding the celebrations of PrEP provision at the time (Nandwani, 2017). Most participants who worked with Black African communities described simply not seeing themselves and their communities as part of PrEP:

You know when [PrEP] was launched I was watching BBC actually and they, BBC Scotland, went out to interview a lot of young gay men who were talking about PrEP and how it’s going to change their lives. There was not even a single woman there and that was publicity for the gay men, that was not publicity for women, regardless of colour, just what they were actually saying when that article came out on television was that “this is PrEP, this is for gay men, now gay men you can access this and this is for you”. There was not a single mention about women because I was watching it. There was nothing to show that actually women it can work.

(Community Practitioners Focus Group)

These public-facing gendered images of PrEP users signalled to those supporting communities of Black African women in Scotland that PrEP was a bio-technology suitable for use with only certain gendered bodies.

Scottish PrEP eligibility criteria at the time of provision did include an ‘all-encompassing’ category that was intended to be inclusive of diverse genders of PrEP candidates: “*Individuals, irrespective of gender, at an equivalent highest risk of HIV acquisition, as agreed with another specialist clinician*.” (Nandwani et al., 2016) However, the active consideration of PrEP as a gendered technology appeared limited in practice to its use by cis-gay and bisexual men and the risk narratives outlined above. This was a particular issue of concern when it came to participants’ reflections about *who* would actually be considered eligible for PrEP. More precisely, they raised concerns around how the eligibility criteria did not adequately anticipate the risk narratives that the women they worked with would need to convey to access PrEP. In discussing the PrEP messaging that described potential candidates as those who ‘might be at risk’, participants perceived a mismatch between what was clinically considered a ‘risk’ and how this might be received within a community setting:

Where you can actually get those people who are vulnerable, because a lot of people, you know, there’s a statement here says “you think you might be at risk”, believe you me, if I was not a woman living with HIV and I was to read that as a 20 year old [laugh] I would look at them and think “what are they talking about, they think I’m promiscuous, I’m not promiscuous” and I would throw it there and forget about it.

(Community Practitioners Focus Group)

While there is a long history of exploring how public health messaging can miss the mark when it comes to effectively communicating with and reaching communities ‘at risk’ (Guttman & Salmon, 2004), participants conveyed how ideas about PrEP users were premised on a particular gendered and clinical recognition of risk to HIV.

Drawing on their community work, participants described how risk of HIV for some women was linked to infidelity on the part of their partners, or in some cases violence or coercion that might stop them from using condoms or other risk reduction strategies within their sexual relationships. One of the primary concerns of participants working with women in this study was that the PrEP narratives that were more likely to be presented by Black African women would not result in accessing PrEP but initiate other responses:

How does that conversation go, like, yeah if someone needs [PrEP] for that sort of protection they’re not going to go to the [local sexual health clinic and say] “so I’m in a domestically violent relationship and I think he might be cheating on me and I want to stay safe”. They’re not going to put it that way because it’s going to raise other alarms that are then going to be actioned.

(Community Practitioners Focus Group)

This extract is drawn from a discussion that centred on anticipated responses to women presenting for PrEP in sexual health clinics and whether a ‘straightforward’ request was likely to result in PrEP provision. While not all anticipated/imagined scenarios were linked to violence within relationships, participants were concerned that for the women they worked with to convey their need for PrEP, they would need to use ‘believable’ PrEP risk narratives. As such, women’s risk narratives were more likely to be perceived as complex, requiring multiple interventions, which did not necessarily include PrEP. In contrast to the previous section where a community worker encouraged gay men to ‘beef’ up their risk narratives, here participants were concerned that by communicating a ‘believable’ PrEP risk narrative, women—and for the most part this meant Black African women—would be clinically treated differently to gay and bisexual men.

## Discussion

In this article, we have sought to illustrate how normative biosexual citizenship was cast at the start of PrEP provision in Scotland, and in particular how this was imagined by those tasked with clinical PrEP provision and/or community support. We have shown how practitioners navigated ideas around who was deserving of support and access to PrEP in the context of limited resources (and wider structural issues relating to PrEP rollout), interpreted what legitimate risk narratives might look like for different groups and translated gendered, sexualised and racialised risk profiles in the context of PrEP provision. This draws attention to how normative biosexual citizenship was not determined only through meeting a set of clinical criteria and adhering to a prophylaxis regime but cast through normative ideas of essential care, constrained resources, risk narratives and gendered and racialised bodies.

Our research has identified tensions in clinical practice and community support around how essential medicine and care are understood, and the processes used to navigate constrained resources by triaging ‘legitimate’ patients. For many clinical practitioners, normative biosexual citizens were expected to present accordingly, bringing with them appropriate attire, acceptable (and ‘straightforward’) HIV risk narratives and demonstrate an ‘appropriate’ reliance on public services. As highlighted, NHS provision of initially very costly PrEP served to place PrEP services into tension with other services that were understood as equally urgent. At times of resource constraints, then, even these deserving patients were cast by some as ‘taking from’ other patients and services. Our research raises important issues in relation to who ought to benefit from new prevention technologies, if these technologies are ‘essential’ and for whom. Where PrEP was globally heralded as transforming the HIV prevention landscape, its provision and support are significantly shaped locally by resource constraints and normative health provision practices, thus challenging PrEP’s status as an essential medicine. Greene has argued that the considerable expansion of the official essential medicines list—of which PrEP is now included—reflects the flexibility of the very concept (Greene, 2015). We must pay attention, then, to how normative castings in local practice may challenge and/or undermine global claims of essential medicines. Moreover, our findings highlight how the idea of the ‘deserving’ biosexual citizen can shape how essential medicine may—or may not—be apportioned more widely across sexual health, with significant implications for those who may not perform in expected ways in sexual health areas that extend beyond HIV (e.g., Mamo et al., 2022; Sanabira, 2016).

We also identified the ways in which gender, race and sexuality were woven through normative citizenship expectations of PrEP users. Our findings suggest that those who do not ‘fit’ expectations may face additional barriers to convince clinical gatekeepers of their need for PrEP. It has been well documented how communities of white cis-gay men have been the primary beneficiaries of PrEP (HPS, 2019; Young, 2021), and much work is underway in relation to expanding and changing the profile of PrEP users (e.g. Grenfell et al., 2022). Our findings draw attention to how the centring of these normative PrEP recipients appears to be built into the very systems which have been created to provide PrEP; those who do not physically fit within anticipated gendered and racialised categories are more likely to struggle to communicate appropriate (or convincing) risk narratives and access appointments for PrEP, a pattern observed across PrEP provision globally (Smith et al., 2022a). This, we argue, is a fundamental problem in equity of access in both early and current configurations of normative biosexual citizenship and one that is not easily addressed.

Since this research, there have been significant developments in how PrEP is provided and negotiated. Indeed, since PrEP provision began, Scottish PrEP clinics have been oversubscribed, adapting their services accordingly (MacDonald et al., 2021). However, we suggest that these early months of PrEP provision were formative and identify key issues that will continue to affect PrEP access and use. For instance, new generations and modalities of PrEP will bring with them increased costs and increasingly complex provision. PrEP (tenofovir disoproxil emtricitabine or TD-FTC) is now available in generic form and at reduced costs to earlier patented versions, and new generations of PrEP have been developed with reduced side effects. Evidence suggests these are important for those with other health conditions such as impaired kidney function and PrEP users over 40 (Brady et al., 2018). However, it has been reported that these new drugs were (initially) unavailable through the NHS (56 Dean Street, 2021), thereby limiting for whom PrEP might be possible. Moreover, new modalities of PrEP recently approved in the UK such as injectable PrEP—highlighted as having increased benefit for those struggling with access to regular services (Waverley Care, 2022)—are likely to raise further issues around the eligibility—or more precisely deservingness—of patients as the provision is rolled out. In turn, this draws attention back to our questions: What is an essential medicine and who can best craft legitimate narratives of clinical need? Ultimately, issues around cost and resource implications, expectations and anticipations about who is attending for PrEP and who ought to be and continued limited access for cis-women, trans and non-binary people and Black African communities and/or other racialised communities speak to how these issues were embedded at the start of provision and continue to shape access and engagement with PrEP. Indeed, they indicate how access to PrEP will continue to demand enactments of normative biosexual citizenship that may well be at odds with the experiences and needs of communities affected by HIV.

## Data Availability

The data that support the findings of this study are available on request from the corresponding author. The data are not publicly available due to privacy or ethical restrictions.

## Footnotes

1 In this article, we use the term ‘trans’ (an abbreviation of transgender) as an inclusive umbrella term for anyone whose gender identity does not fully correspond with the sex assigned to them at birth. We use the term ‘cis’ to refer to people whose gender fully corresponds with the sex assigned to them at birth.

2 These criteria were primarily based on those used in the PROUD trial (the first UK PrEP trial) which was only open to gay and bisexual men (McCormack et al., 2016; Nandwani et al., 2016). The fourth criteria—meeting an equivalent risk—was introduced so as to account for PrEP users that were not cis-gay and bisexual men and may not meet the same clinical risk profile outlined in the first criteria (Young, 2021).
